# Differential regulation of muscarinic M_1 _receptors by orthosteric and allosteric ligands

**DOI:** 10.1186/1471-2210-9-14

**Published:** 2009-12-02

**Authors:** Christopher N Davis, Stefania Risso Bradley, Hans H Schiffer, Mikael Friberg, Kristian Koch, Bo-Ragnar Tolf, Douglas W Bonhaus, Jelveh Lameh

**Affiliations:** 1 ACADIA Pharmaceuticals Inc, 3911 Sorrento Valley Blvd, San Diego, CA 92121, USA; 2 European Patent Office, Hohenzollernstrasse 150, 80796, Munich, Germany; 3 Genoptix Medical Laboratory, 2110 Rutherford Road, Carlsbad, CA 92008, USA

## Abstract

**Background:**

Activation of muscarinic M_1 _receptors is mediated *via *interaction of orthosteric agonists with the acetylcholine binding site or *via *interaction of allosteric agonists with different site(s) on the receptor. The focus of the present study was to determine if M_1 _receptors activated by allosteric agonists undergo the same regulatory fate as M_1 _receptors activated by orthosteric agonists.

**Results:**

The orthosteric agonists carbachol, oxotremorine-M and pilocarpine were compared to the allosteric agonists AC-42, AC-260584, *N*-desmethylclozapine and xanomeline. All ligands activated M_1 _receptors and stimulated interaction of the receptors with β-arrestin-1. All ligands reduced cell surface binding and induced the loss of total receptor binding. Receptor internalization was blocked by treatment with hypertonic sucrose indicating that all ligands induced formation of clathrin coated vesicles. However, internalized receptors recycled to the cell surface following removal of orthosteric, but not allosteric agonists. Whereas all ligands induced loss of cell surface receptor binding, no intracellular vesicles could be observed after treatment with AC-260584 or xanomeline. Brief stimulation of M_1 _receptors with AC-260584 or xanomeline resulted in persistent activation of M_1 _receptors, suggesting that continual receptor signaling might impede or delay receptor endocytosis into intracellular vesicles.

**Conclusion:**

These results indicate that allosteric agonists differ from orthosteric ligands and among each other in their ability to induce different regulatory pathways. Thus, signaling and regulatory pathways induced by different allosteric ligands are ligand specific.

## Background

Muscarinic M_1 _receptors are the predominant muscarinic receptor subtype expressed in the brain with high levels of expression in the cerebral cortex and hippocampus. Muscarinic M_1 _receptors are believed to mediate many important central processes such as cognition and memory [1,2] and thus are targeted for the development of drugs to treat various neurological disorders, such as Alzheimer's disease. However, discovery of selective M_1 _agonists has been challenging due to the highly conserved orthosteric acetylcholine binding site of muscarinic receptors. Recent structure-function studies have led to the identification of functionally selective muscarinic M_1 _agonists [3-8]. Mutagenesis studies have shown that mutations that abolish activation of M_1 _receptors by orthosteric ligands do not affect, and in some cases even enhance, activation by these selective ligands [4,9]. These novel selective ligands have been named allosteric M_1 _agonists because their mode of interaction with M_1 _receptor is different from that of orthosteric agonists [4,10-12].

While allosteric M_1 _agonists appear to bind to a receptor domain different from that of the orthosteric ligands, in many cases they have been shown to induce similar signaling pathways as the orthosteric M_1 _agonists. Moreover, allosteric M_1 _agonists can displace conventional orthosteric M_1 _muscarinic radioligands such as [^3^H]-NMS [4,12-14]. Based on these data, it has been suggested that there is some degree of overlap between the allosteric and orthosteric binding sites on the M_1 _receptor for these ligands [12].

A recent study [14] has demonstrated that some allosteric muscarinic M_1 _agonists differentially activate downstream signaling pathways for M_1_receptors, suggesting "stimulus trafficking" with respect to receptor signaling events. However, presently little is known about the potential differences between orthosteric and allosteric muscarinic M_1 _ligands in modulating receptor regulatory pathways such as internalization, down-regulation or recycling. The present study was carried out to assess these cellular regulatory events in muscarinic M_1 _receptors in response to a number of allosteric and orthosteric M_1 _agonists.

In order to understand the mode of interaction of a ligand with the receptor, it is important to characterize receptor-ligand interaction in multiple assays and in multiple systems [12] and also to correlate it with the induction of downstream signaling and regulatory pathways. Receptor regulatory events include receptor processes such as desensitization (rapid uncoupling of receptors from signaling molecules), internalization/endocytosis (translocation of receptors from the cell surface into intracellular vesicles) and down-regulation (translocation of receptors into lysosomes where receptors are degraded).

In the present study, several different assays were employed in order to comprehensively evaluate various intracellular signaling and regulatory processes. The results demonstrate that allosteric ligands differ from orthosteric ligands and amongst each other in induction of internalization, down-regulation, recycling and signaling of M_1 _muscarinic receptors.

A preliminary report of the present work was presented at the meetings for Society for Neuroscience, 2007 and at Recent Advances in Muscarinic Receptor Pharmacology and Therapeutics Colloquium, 2008.

## Results

### Allosteric and orthosteric agonists induce signaling of human muscarinic M_1 _receptors

Receptor activation in response to agonist treatment was measured by two different assays, Receptor Selection and Amplification assay (RSAT) and phosphatidylinositol (PI) hydrolysis assay (Table 1). These two assays showed similar results for all compounds tested. Among the ligands tested, AC-42 and NDMC were shown to be partial agonists in both assays, while all the other compounds were able to activate M_1 _receptor with full efficacy as compared to carbachol.

**Table 1:** Activation of muscarinic M_1 _receptors as measured by R-SAT^® ^and PI assays

	M_1 _Agonist RSAT		PI Hydrolysis	
	**pEC**_50_	% Efficacy	**pEC**_50_	% Efficacy
**Carbachol**	6.2 ± 0.3	100 ± 10	6 ± 0.1	100 ± 3
**Oxotremorine-M**	7.4 ± 0.3	105 ± 13	7.0 ± 0.1	106 ± 7
**Pilocarpine**	5.7 ± 0.2	93 ± 15	6.2 ± 0.9	97 ± 31
**Xanomeline**	7.4 ± 0.5	120 ± 21	8.4 ± 0.2	99 ± 12
**NDMC**	7.4 ± 0.2	76 ± 13	7.1 ± 0.4	67 ± 23
**AC-42**	6.7 ± 0.3	62 ± 14	6.5 ± 0.5	79 ± 14
**AC-260584**	7.6 ± 0.4	102 ± 21	7.7 ± 0.3	90 ± 17

Activities of compounds on human M_1 _receptors were compared using two different assay; RSAT in transiently transfected NIH-3T3 cells and PI hydrolysis in stable CHO-M_1 _cells. Data for each compound is normalized to carbachol for both assays. Values presented are mean ± SD from 2-19 independent experiments carried out in triplicate.

### Allosteric M_1 _agonists, AC-260584 and xanomeline, show distinctive effects on receptor endocytosis

**Figure 1:**
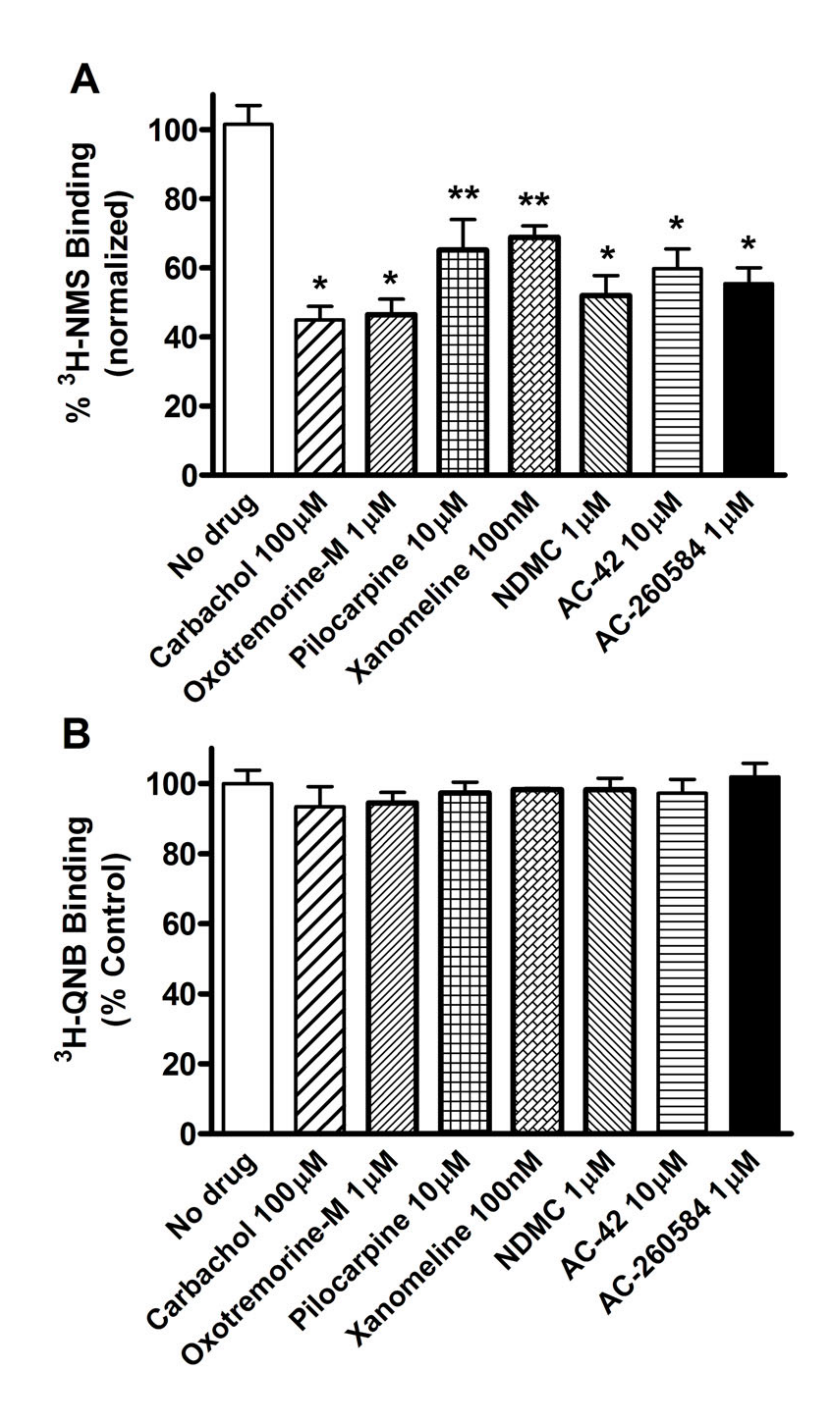
**Loss of binding to cell-surface receptors following treatment with orthosteric and allosteric ligands**. Binding was measured in transiently-transfected HEK-293 cells. (A) Cell surface receptor binding was assessed with the [^3^H]-NMS and (B) total receptor binding was assessed with [^3^H]-QNB as detailed in Methods. Surface binding was normalized to total receptor binding and expressed as % of control (no drug). Average receptor expression in untreated transiently transfected HEK cells was 325,000 sites/cell as measured by [^3^H]-NMS and 360,000 site/cell as measured by [^3^H]-QNB. Figure shows the mean results (± S.D.) from test compounds assayed in triplicate in a representative experiment repeated 2-6 times. Cell surface receptors remaining after treatment with each compound were as listed; No drug, 102% ± 13, carbachol, 45% ± 10, oxotremorine-M, 46% ± 8, pilocarpine, 65% ± 12, xanomeline, 69% ± 5, NDMC, 52% ± 10, AC-42, 60% ± 14, AC-260584, 55% ± 11. Statistically significant difference compared to no drug, * = p < 0.001, ** = p < 0.05 (Student t-test, Graph-Pad, Prism).

Treatment with all the orthosteric and allosteric M_1 _agonists resulted in reduced cell surface binding (Figure 1A) as measured with [^3^H]-NMS, while having no effect on total receptor binding as measured with [^3^H]-QNB (Figure 1B).

**Figure 2:**
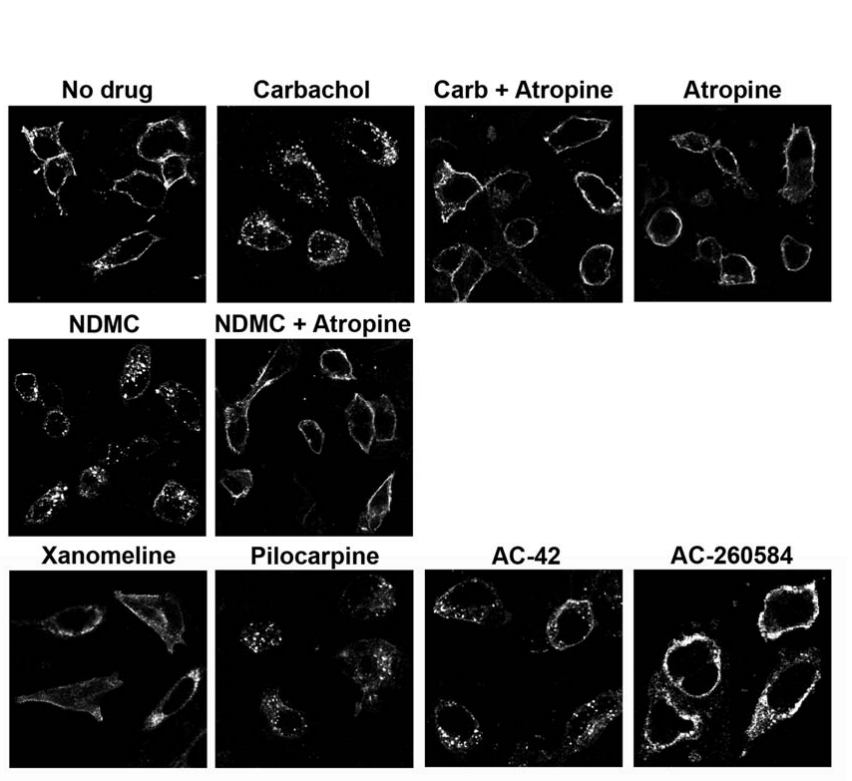
**Localization of M_1 _receptors after treatment with various ligands as visualized by confocal microscopy**. Cells were treated with various compounds (1 mM carbachol, 1 μM oxotremorine-M, 10 μM pilocarpine, 1 μM xanomeline, 10 μM NDMC, 10 μM AC-42 or 10 μM AC-260584) for 2 hrs at 37°C in the absence or presence of 100 nM atropine. The agonist concentrations used were chosen to ensure maximum activation of endocytic pathway. These concentrations were higher than those used in binding assays. However the possibility of residual ligand confounding the results was not a concern since the site of interaction with the monoclonal antibody (N-terminal tail of the receptor) is distinct from the agonist binding sites. Images are from a representative experiment repeated 3 times with similar results.

To visualize endocytosis of M_1 _receptors from the cell surface into endosomes, translocation of the M_1 _receptors was followed using confocal microscopy. The results demonstrated that treatment with carbachol, pilocarpine, oxotremorine-M, NDMC and AC-42 induced translocation of cell surface receptors into intracellular vesicles, while no receptors could be visualized in the endosomes after treatment with AC-260584 or xanomeline (Figure 2). Translocation of M_1 _receptors seen with these ligands was blocked by treatment with the muscarinic antagonist atropine (Figure 2).

**Figure 3:**
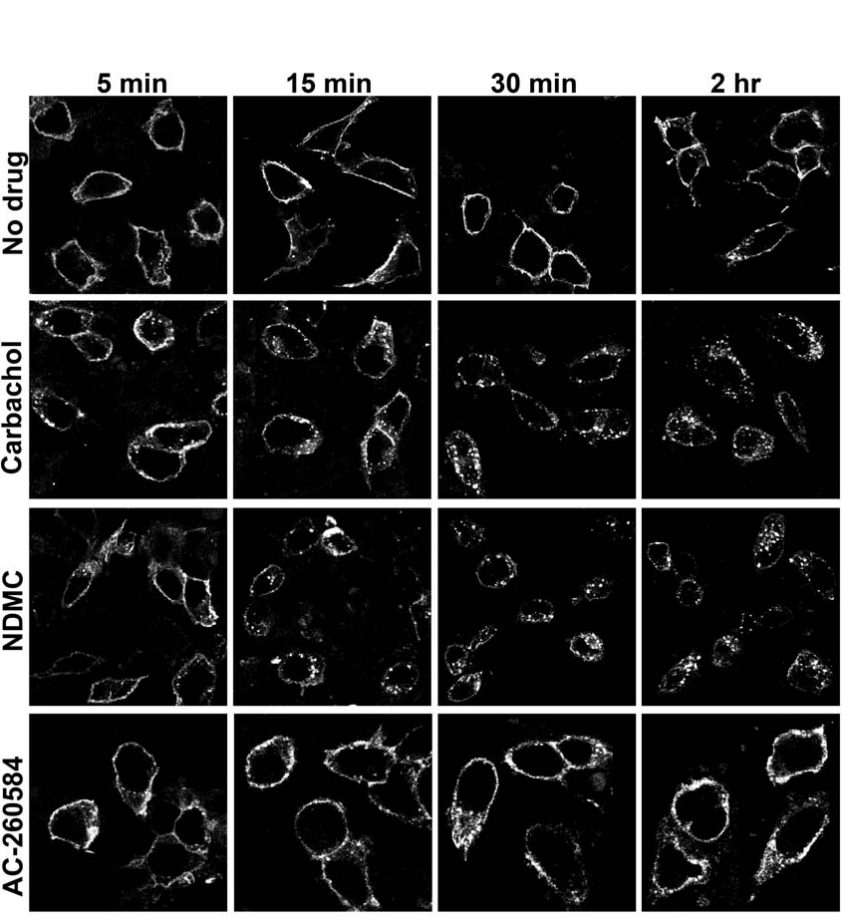
**Time course of receptor internalization as visualized by confocal microscopy**. Translocation of cell surface receptors into intracellular vesicles was measured for carbachol (1 mM), NDMC (10 μM) and AC-260584 (10 μM). Images are from a representative experiment repeated 2-3 times with similar results.

The time course of receptor internalization was assessed for some of these compounds. Carbachol and NDMC induced translocation of receptors into intracellular vesicles within five minutes of treatment, while AC-260584 did not induce formation of any vesicles even after 2 hours of treatment (Figure 3).

### Hypertonic sucrose treatment blocks M_1 _receptor internalization

**Figure 4:**
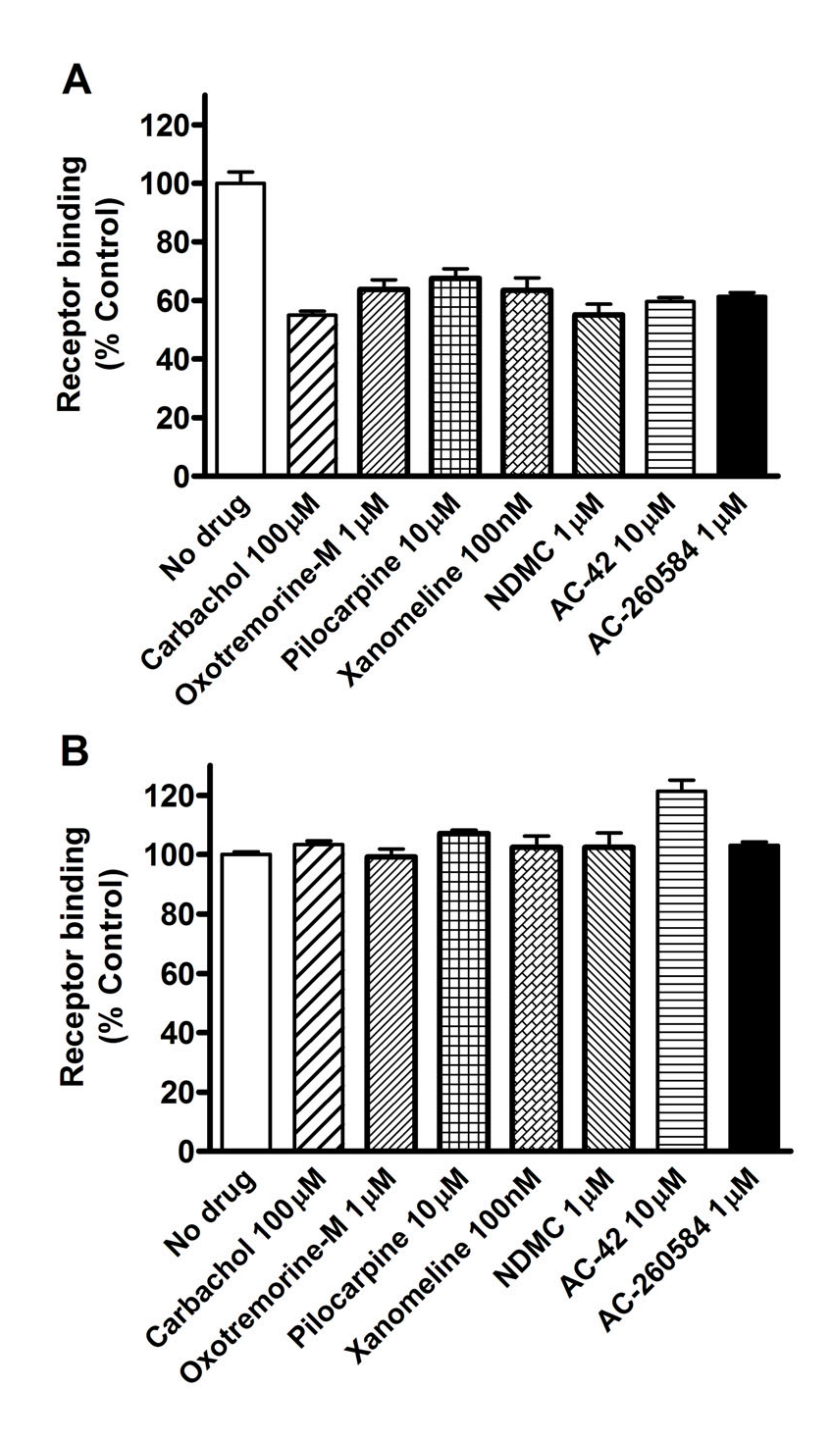
**Treatment with hypertonic sucrose blocked internalization induced by all ligands studies**. Cells were treated without (A) or with (B) 0.4 M sucrose during treatment with the various ligands at indicated concentrations. Receptors remaining at the cell surface were quantitated. Graphs are mean results (± S.D.) from test compounds run in triplicate in a representative experiment repeated 2 times with similar results.

Treatment of the HEK-293 cells with hypertonic sucrose blocked loss of cell surface receptor binding induced by all allosteric and orthosteric M_1 _agonists (Figure 4B). These results indicate that the loss of cell surface receptors induced by all these compounds are mediated via a common cellular pathway, namely by formation of clathrin-coated pits.

### Lack of M_1 _receptor recycling following treatment with allosteric agonists

Receptor recycling to the cell surface following removal of the agonists was assessed in HEK-293 cells transiently transfected with hM_1_. Agonist removal after treatment with carbachol and oxotremorine-M resulted in recovery of cell surface binding as measured by [^3^H]-NMS. However, no receptor recycling could be observed following treatment with the allosteric agonists, AC-42 and AC-260584 or after treatment with xanomeline. The extent of recovery of [^3^H]-NMS binding following removal of each ligand is summarized in Table 2.

**Table 2:** Recycling of muscarinic receptors after treatment with various ligands

	No recycle	3 h recycle
**No drug**	100	100
**Carbachol**	48 ± 4*	102 ± 10
**Oxotremorine-M**	60 ± 2*	97 ± 1
**Xanomeline**	65 ± 5*	69 ± 7*
**AC-42**	63 ± 3*	69 ± 1*
**AC-260584**	59 ± 2*	64 ± 3*

HEK-293 cells expressing hM_1 _were treated with various ligands for 30 minutes as indicated in the table. Concentrations of ligands used were as follows; carbachol, 100 μM; oxotremorine-M, 1 μM; xanomeline, 100 μM; AC-42, 10 μM; and AC-260584, 1 μM and similar to concentration used in the internalization assays. Values presented are % receptors on the cells surface. Data presented is the average from 2 independent experiments carried out in triplicate. Statistically significant difference compared to no drug, * = p < 0.05, (Student t-test, GraphPad, Prism).

No recycling could be observed after treatment with NDMC (1 μM), 71% cell surface receptor after 3 hours of recycling (n = 1).

### Allosteric and orthosteric M_1 _agonists induce down-regulation of M_1 _receptors in CHO-M1 cells

**Figure 5:**
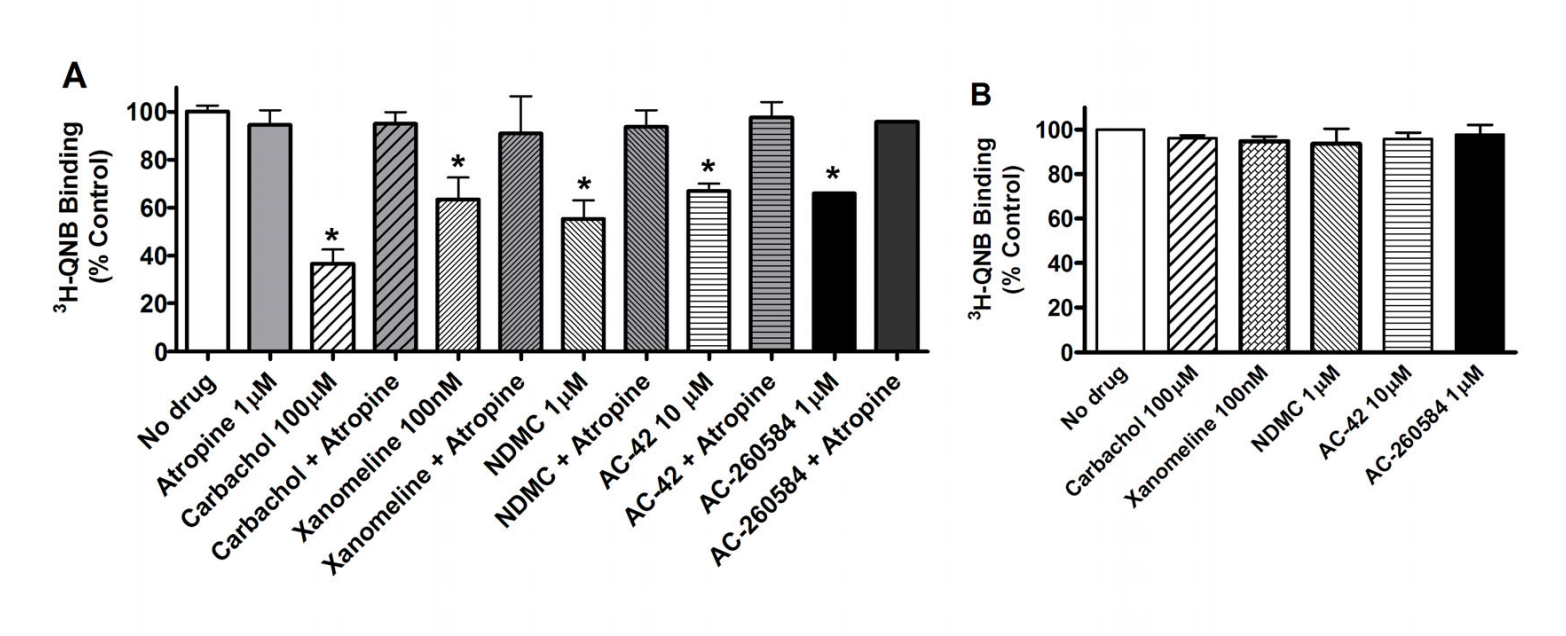
**Down-regulation of Muscarinic M_1 _Receptors in CHO Cells**. (A) Total receptor binding remaining following 24 hours of treatment with various agonists, in the presence or absence of the muscarinic antagonist atropine, was measured in CHO cells stably expressing hM_1 _receptors. The data presented are mean ± S.D from 3-6 pooled experiments performed in triplicate. Total binding is expressed as % of control at 24 hours. Total receptor binding remaining after treatment with each ligand was; No drug, 100% ± 3, carbachol, 49% ± 3, xanomeline, 48% ± 12, NDMC, 49% ± 6, AC-42, 56% ± 7, AC-260584, 56% ± 4. Statistically significant difference compared to no drug, * = p < 0.001, (Student t-test, GraphPad, Prism). (B) No loss in [^3^H]-QNB binding could be measured when the cells were treated with the same concentration of each ligand for only 2 hours, confirming proper removal of ligands during washing.

The extent of down-regulation of M_1 _receptors following treatment with various ligands was measured in CHO cells stably expressing hM_1 _receptors. Treatment of the cells with all the orthosteric and allosteric ligands for 24 hours resulted in a loss of total receptor binding (Figure 5A). Treatment with similar concentrations of the agonists for 2 hours did not result in any loss of [^3^H]-QNB binding, confirming proper removal of ligands from the cells (Figure 5B). Receptor down-regulation induced by each of these ligands was mediated by interaction with the M_1 _receptors, as it could be blocked by treatment with the muscarinic antagonists atropine (1 μM) (Figure 5A), scopolamine (1 μM) or pirenzepine (10 μM) (data not shown).

### Allosteric and orthosteric M_1 _agonists induce recruitment of β-arrestin-1 to M_1 _receptors

The ability of different muscarinic agonists to induce association of the M_1 _receptors with β-arrestin-1 in real time was quantified using the BRET-2 assay. Most ligands were able to induce an association of the receptor with β-arrestin-1, albeit with different potencies and efficacies (Table 3). Relative efficacies of the compounds were determined as compared to carbachol. Oxotremorine-M was a full agonist in this assay, while pilocarpine, xanomeline, AC-260584, and AC-42, were partial agonists with relative efficacies in the range of 12-26%. NDMC did not show any agonist activity at M_1 _as measured by BRET-2.

**Table 3:** Activation of muscarinic M_1 _receptors as measured by BRET-2

	BRET-2	
Compound	**pEC**_50_	%Efficacy
**Carbachol**	4.6 ± 0	100 ± 0
**Oxotremorine-M**	5.6 ± 0.2	100 ± 12
**Pilocarpine**	5.4 ± 0.3	16 ± 3*
**NDMC**	NA	
**AC-42**	5.4 ± 0.6	12 ± 0.7*
**AC-260584**	6.2 ± 0.14	26 ± 6*
**Xanomeline**	7.2 ± 0.3	16 ± 6*

M_1 _BRET-2 assays were performed to determine potency and efficacy of agonists to stimulate β-arrestin-1 recruitment. Data presented are mean ± S.D. from 2-3 independent experiments carried out in triplicate. Statistically significant difference compared to no drug, * = p < 0.001, (Student t-test, GraphPad, Prism). Based on student t-test, efficacies for AC-42, AC-260584, xanomeline and pilocarpine were not statistically different, p > 0.5. NA: No measurable agonist activity.

### M_1 _receptor endocytosis is distinctively affected by AC-260977 an AC-260584 related ligand

**Figure 6:**
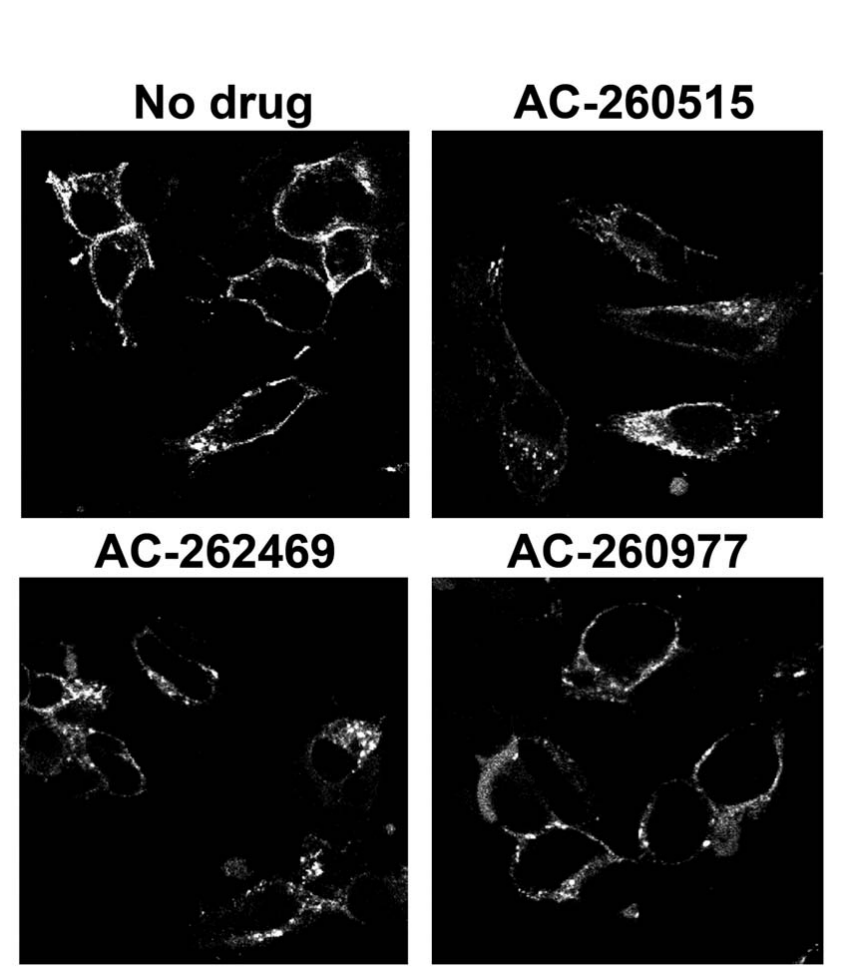
**Internalization of muscarinic M_1 _receptors by analogues of AC-260584**. HEK-293 cells expressing EE-hM_1 _were treated with 1 μM of each ligand and localization of EE-hM_1 _receptors were visualized using confocal microscopy. Images are from a representative experiment repeated 2-3 times with similar results.

To determine whether the lack of induction of receptor endocytosis observed with AC-260584 was unique to this compound or to other compounds with similar structure, the effects of three closely related compounds with various activities (Table 4) on receptor internalization were assessed. While two of these compounds induced translocation of the receptors from cell surface into intracellular vesicles in HEK-293 cells, one compound, AC-260977, did not (Figure 6). This data indicates that even compounds with close structural similarity to AC-260584 can behave differently with respect to inducing receptor endocytosis.

**Table 4:** Activation of muscarinic M_1 _receptors by analogues of AC-260584 as measured by R-SAT

	M_1 _Agonist RSAT	
	**pEC**_50_	%Efficacy
**AC-260515**	6.6 ± 0.2	66 ± 16
**AC-262469**	7.2 ± 0	107 ± 16
**AC-260977**	7.7 ± 0.1	107 ± 10

Activities of AC-260584 analogues on human M_1 _receptors were assessed using R-SAT in transiently transfected NIH-3T3 cells. Data presented are mean ± SD from 5-7 independent experiments carried out in triplicate.

### AC-260584 and xanomeline induce persistent activation of M_1 _receptors

**Figure 7:**
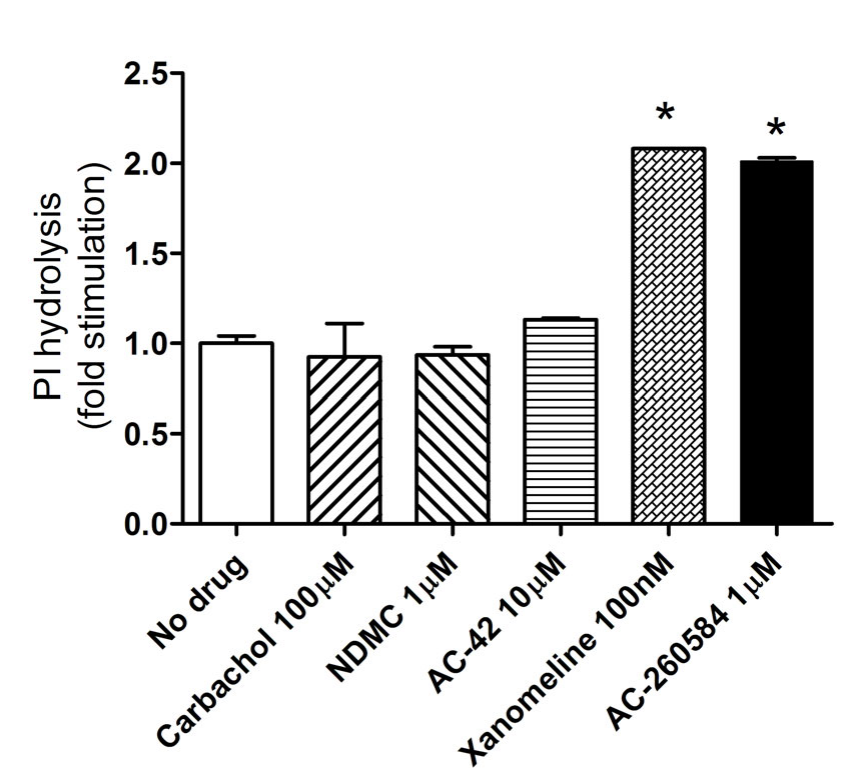
**Continued activation of M_1 _receptors after agonist treatment**. CHO-hM_1 _cells were treated with agonists for 1 minute prior to washing and incubation in the presence of 10 mM LiCl_2_. Total IP accumulation after 1 hour was measured as described under Methods. Data presented are mean ± SD for duplicate measurements from a representative experiment repeated twice with similar results. Statistically significant difference compared to no drug, * = p < 0.05, (Student t-test, GraphPad, Prism).

To investigate other ligand-mediated receptor modifications, changes in receptor coupling were assessed in CHO-M_1 _cells treated with various ligands. Cells were briefly exposed to each agonist and PI accumulation was measured following removal of the agonist. Basal levels of PI hydrolysis were elevated after only one minute of exposure to AC-260584 and xanomeline. Elevation of basal receptor activity was not seen following treatment with carbachol, NDMC or AC-42 (Figure 7). These data suggest that only one minute of treatment with AC-260584 and xanomeline can induce a modification in the receptor conformation resulting in continuous signaling of the receptor in the absence of continued presence of the agonist. This increased basal activity of the receptors following treatment with AC-260584 and xanomeline was partially blocked in the presence of increasing concentrations of the inverse agonist atropine (data not shown).

## Discussion

The present study was designed to characterize various receptor regulatory pathways initiated after treatment with allosteric or orthosteric M_1_agonists. We demonstrate that specific regulatory pathways initiated after activation of M_1 _receptors with different ligands are unique to each ligand and not due to the allosteric or orthosteric nature of receptor-ligand interaction.

Among the M_1 _agonists in the present study, AC-42 and AC-260584 have been characterized as allosteric agonists [4,12,15]. Other compounds, NDMC and xanomeline, were reported to have an allosteric component in their interaction with M_1 _receptors [4,9,13,16]. Carbachol, oxotremorine-M and pilocarpine are classical orthosteric agonists.

All agonists in this study were characterized as either full agonists or high efficacy partial agonists at M_1 _receptors as measured by RSAT and PI assays. No correlation could be made between efficacy or potency of each agonist and its efficacy in activation of various receptor regulatory processes. Moreover, none of the agonists studied induced receptor desensitization, as measured by the lack of a shift in carbachol dose response curve or a change in maximal response in PI hydrolysis assay (data not shown). Still, the possibility of desensitization of other signaling pathways cannot be ruled out.

To assess the differences in receptor regulatory processes induced by each agonist, the extent of loss of cell surface receptors and total receptors were evaluated. All ligands were capable of inducing a loss of receptor binding. These results indicate that the processes of receptor internalization and down-regulation are induced after treatment with all these ligands.

To further assess receptor internalization/endocytosis, confocal microscopy was used to visualize receptor localization. After treatment with orthosteric ligands, carbachol, pilocarpine and oxotremorine-M, M_1 _receptors could be visualized in endosomes. However, after treatment of the cells with the allosteric agonist AC-260584, no receptors could be observed in the endosomes. To test the hypothesis that allosteric interaction with the receptors was responsible for this difference, we tested several other allosteric agonists for their propensity to induce receptor endocytosis into endosomes. The allosteric ligands AC-42 and NDMC were able to induce receptor internalization/endocytosis, while xanomeline was not. To determine whether lack of endocytosis was associated with the specific structure of AC-260584, three close analogues of this compound were studied. Two of these compounds induced internalization/endocytosis of M_1 _receptor while one did not. These results indicate that allosteric nature of the ligand-receptor interaction is not sufficient explanation for lack of ligand-induced endocytosis.

It was puzzling that no intracellular vesicles could be seen after treatment with AC-260584 and xanomeline while binding to cell surface receptors was reduced after treatment with these ligands. One possible reason for this observation could be induction of different endocytic pathways by these ligands. It has been previously demonstrated that carbachol-induced internalization of M_1 _receptors is mediated via clathrin-coated pits [17]. Formation of clathrin-coated vesicles can be blocked by treatment with hypertonic sucrose solution [18-21]. In order to investigate whether AC-260584 and xanomeline induce similar endocytic pathways as carbachol, cells were pretreated with hypertonic sucrose solution to block formation of clathrin coated vesicles. Hypertonic sucrose treatment blocked receptor internalization induced by all the agonists, indicating that formation of clathrin coated vesicles is necessary for the loss of cell surface receptor binding after treatment with all these ligands. This observation suggests that divergence of receptor regulatory processes induced by the different ligands occur down-stream from the formation of clathrin-coated pits.

Internalization of G-protein coupled receptors has been shown to involve interaction with β-arrestin [22-27]. Moreover, it has been suggested that endocytosis and signaling of muscarinic M_1 _receptors involve interaction with β-arrestin [23,24,26]. In order to determine if absence of interaction with β-arrestin attributed towards the differences observed in vesicle formation, interaction of M_1 _receptors with β-arrestin-1 was assessed using BRET-2 assays. All ligands studied were able to stimulate recruitment of β-arrestin-1 to M_1 _receptors. While carbachol and oxotremorine-M were full agonists in BRET-2 assay, all other ligands, including pilocarpine, were partial agonists. Other studies have also described pilocarpine as a partial agonist at M_1 _receptors [28,29]. Although AC-260584 and xanomeline stimulated an increase in the BRET-2 signal with lower efficacy compared to carbachol and oxotremorine-M, the lower efficacy in this interaction could not explain the absence of vesicle formation in response to these ligands. This conclusion is based on the fact that while both AC-42 and pilocarpine have similar potency and efficacy in the BRET-2 assay compared to AC-260584 and xanomeline, both compounds can induce receptor endocytosis into endosomes.

Another difference observed between the receptor regulatory processes induced by the different agonists was the difference seen in the recycling of the receptors back to the cell surface. While M_1 _receptors recycled back to the cell surface after treatment with orthosteric ligands (carbachol and oxotremorine-M) following treatment with allosteric ligands (AC-260584, AC-42, xanomeline or NDMC) and ligand removal, binding to cell surface receptors remained low. These results suggest that after removal from the cell surface, receptors sequestered following treatment with the latter set of compounds do not return to the cell surface. We have shown that after a brief treatment with some of these ligands, M_1 _receptors remain active and produce higher basal levels of inositol phosphates. Thus, it is likely that receptors treated with these ligands remain in a sequestered state and continue to signal via G-proteins (AC-260584 and xanomeline) or other signaling proteins. Following this continued signaling event, the receptors are then processed through various degradative processes and subsequently destroyed.

**Figure 8:**
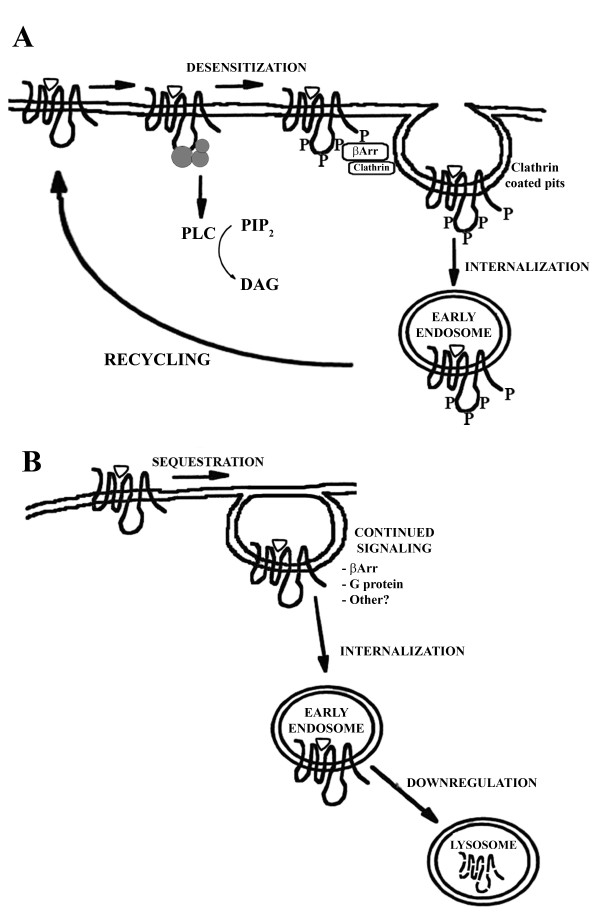
**Various receptor signaling and regulatory pathways**. (A) Receptor activation by agonist leads to rapid phosphorylation, interaction with β-arrestin and sequestration into clathrin-coated pits. Coated pits containing the receptors pinch off the plasma membrane into intracellular vesicles. Internalized receptors are then de-phosphorylated and recycled back to the cell surface for further stimulation or are routed to the lysosomes for degradation (down-regulation). (B) Following treatment with some agonists it is possible that receptors do not recycle back to the surface, implying that these receptors remain sequestered and continue to signal before being routed to the lysosomes for degradation.

Based on the classical paradigm for GPCR regulation, receptor activation by agonist leads to rapid phosphorylation, interaction with β-arrestin and sequestration into clathrin-coated pits. Coated pits containing the receptors pinch off the plasma membrane into intracellular vesicles via a dynamin mediated process. Internalized receptors are then de-phosphorylated and recycled back to the cell surface for further stimulation (Figure 8A) or are routed to the lysosomes for degradation, a process known as down-regulation [27]. Our results indicate that activation of M_1 _receptors by allosteric ligands do not stimulate the recycling pathway in HEK-293 cells. Absence of receptor recycling by these ligands implies that M_1 _receptors stimulated by these agonists remain sequestered and continue to signal or are routed to the lysosomes for degradation (Figure 8B). In addition to its role in receptor regulation, receptor endocytosis has been implicated in signaling of GPCRs [30,31]. Signaling pattern of M_1 _receptors following treatment of the cells with various agonists was assessed. Interaction of M_1 _receptors with caveolin after treatment with various ligands was evaluated using confocal microscopy. As seen previously for carbachol [17], no co-localization of M_1 _receptors with caveolin was observed after treatment with any of the allosteric agonists studied (data not shown). The possibility of receptors continuing to signal after treatment with some ligands and not others was assessed by measuring inositol phosphates following only 1 minute of treatment with the ligands. While no changes in basal levels of PI hydrolysis could be observed after treatment with carbachol and oxotremorine-M, treatment with AC-260584 and xanomeline resulted in increased basal levels of inositol phosphates. These results suggest that treatment with these compounds could render a conformational change in the receptor that may possibly lead to continued signaling of M_1 _receptors.

**Figure 9:**
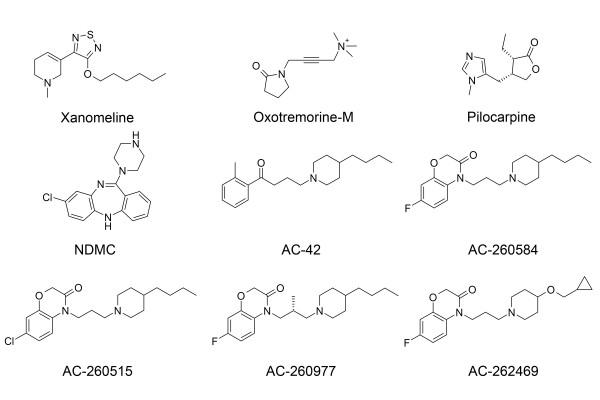
**Structures of various compounds**. Structures of the orthosteric and allosteric muscarinic agonists characterized in the present study are represented.

Based on the results of these studies, it may be more appropriate to classify the agonists studied (Figure 9) on the basis of their activation of various cellular processes rather than by the mode of interaction with various domains of the receptor. Thus, we can classify these ligands as follows; (1) carbachol and oxotremorine-M fully activate all processes investigated. (2) Pilocarpine can fully activate all processes except it is a partial agonist in BRET-2 assay. (3) NDMC and AC-42 are partial agonists of various signaling pathways of M_1 _receptors; fully activate internalization and down-regulation of the receptors, but not receptor recycling processes. (4) AC-260584 and xanomeline are full agonists at M_1 _as assessed by RSAT and PI, but are partial agonists in BRET-2 assay. Both these compounds induce loss of cell surface and total receptors, but no recycling or vesicle formation can be measured after treatment with these compounds. Moreover, treatment with these compounds can possibly induce continued signaling of M_1 _receptors. These last two compounds have the most diverse mode of interaction with M_1 _receptors compared to all other agonist studied.

## Conclusion

The results from the present study indicate that M_1 _signaling and regulatory pathways induced by different allosteric M_1 _agonists are ligand specific. Allosteric agonists differ from orthosteric agonists and amongst each other in their ability to induce different regulatory processes for M_1 _receptors. Moreover, it is important to note that although there are differences in how multiple types of agonists can regulate the activity of M_1 _receptors, activation with allosteric ligands, like activation with orthosteric ligands, ultimately results in receptor internalization/down-regulation. Thus, it is expected that the normal physiological limits on receptor signaling will occur regardless of which agonist is used.

## Methods

### Materials

Human embryonic kidney (HEK-293) cells were purchased from American Tissue Culture Collection. Chinese hamster ovary (CHO) cells stably expressing human muscarinic M_1 _receptors (CHO-M_1_) were established as described previously [32]. [^3^H]-NMS and [^3^H]-QNB were purchased from GE Healthcare (Piscataway, NJ). Carbachol, atropine, oxotremorine-M, pilocarpine and poly-D-lysine were purchased from Sigma-Aldrich (St. Louis, MO). Xanomeline, *N-*desmethylclozapine (NDMC), AC-260584 (4-[3-(4-butylpiperidin-1-yl)propyl]-7-fluoro-2*H-*benzo[*b*][1,4]oxazin-3-one)), AC-42 (4-(4-butylpiperdin-1-yl)-1-(2-methylphenyl)butan-1-one), AC-260515 (4-[3-(4-butyl-piperidin-1-yl)-propyl]-7-chloro-2*H-*benzo[*b*][1,4]oxazin-3-one), AC-262469 (4-[3-(4-cyclopropylmethyloxypiperidin-1-yl)propyl]-7-fluoro-2*H-*benzo[*b*][1,4]oxazin-3-one) and AC-260977 ((*R*)-4-[3-(4-butylpiperidin-1-yl)propyl]-7-fluoro-2*H-*benzo[*b*][1,4]oxazin-3-one) were synthesized at ACADIA Pharmaceuticals LLC. The EE-hM_1 _antibody was prepared as described previously [33]. All other reagents were obtained from common vendors.

### Receptor binding assays

Two different systems were employed to assess receptor internalization and down-regulation in response to various ligands. This selection was based on previous data identifying each system as the best choice for the specific study.

Previous studies have shown that transiently transfected HEK-293 cells efficiently internalize M_1 _muscarinic receptors [34,35] while CHO cells do not [21]. To quantify receptor internalization, agonist-induced decreases in cell surface receptor binding were measured in HEK-293 cells transiently transfected with hM_1 _receptor by using non cell-permeating quaternary amine, [^3^H]-NMS. In addition, in each case total receptor binding was measured by the membrane permeating tertiary amine, [^3^H]-QNB. In each case, the highest concentration of agonist that did not result in a reduction of total receptor binding after 2 hours, as measured by [^3^H]-QNB, was selected for the assay. Using this process for the selection of agonist concentration ensured that all residual ligand had been completely removed and could not contribute to reduction of receptor binding.

Previous studies have demonstrated that after chronic treatment with the muscarinic agonist carbachol, hM_1 _receptors stably expressed in HEK-293 cells do not efficiently go through receptor down-regulation process [34] while CHO cells are a good model to study this regulatory pathway [21,36]. Thus, in order to assess receptor down-regulation, loss of total receptor binding was measured in stably transfected CHO cells using [^3^H]-QNB. CHO-M_1 _cells were grown in F-12 medium supplemented with 10% fetal bovine serum.

HEK-293 cells were grown in DMEM medium supplemented with 10% fetal bovine serum. For studies using transiently transfected cells, exponentially growing HEK-293 cells (6 × 10^6 ^cells) were seeded onto 10 cm tissue culture dish and transfected with hM_1 _in PSI vector using FuGene HD transfection reagent (Roche Applied Sciences, Indianapolis, IN) following manufacturer's instructions. One day after transfection, the cells were harvested using phosphate buffered saline (PBS) containing EDTA and seeded onto 24-well tissue culture plates as described below.

For receptor binding studies, cells were seeded onto 24-well tissue culture plates (poly D-lysine coated for HEK-293 cells), allowed to attach overnight, and then treated with various concentrations of the compounds in serum-free medium at 37°C for 2 hrs (internalization studies, HEK-M_1_) or 24 hrs (down-regulation studies, CHO-M_1_). Cells were placed on ice, washed three times with ice-cold PBS, and incubated with PBS containing saturating concentration of [^3^H]-NMS (2 nM, internalization assay) or [^3^H]-QNB (1 nM, down-regulation assays) at 12°C for 90 min [21,34]. These conditions were established previously and were sufficient for achieving equilibrium binding [21,34]. Binding assays were carried out at 12°C to ensure no receptor recycling occurred. Cells were placed on ice, scraped, harvested by filtration (24-well GF/B plates, Perkin Elmer, Waltham, Massachusetts), and washed three times with ice-cold PBS. The radioactivity on the filters was quantitated by liquid scintillation counting using Top Count (Perkin Elmer, Waltham, Massachusetts). Average hM_1 _receptor expression in untreated transiently transfected HEK cells was 325,000 sites/cell as measured by [^3^H]-NMS and 360,000 site/cell as measured by [^3^H]-QNB. Average receptor expression in untreated CHO-M_1 _cells was 280,000 sites/cells as measured by [^3^H]-QNB. Thus receptor expression levels were roughly similar in both these cells lines.

### Recycling experiments

HEK-293 cells transiently transfected with hM_1 _receptors were treated with various ligands for 30 minutes at 37°C. Following treatment with the ligands, control (non-recycle) plates were washed with ice-cold PBS and 2 nM [^3^H]-NMS in PBS was added for 90 min at 12°C. Recycling plates were washed 3× with room temperature PBS. Serum free media was added and cells were incubated for 3 hrs at 37°C. After this incubation period, presence of receptors at the cell surface, as measured by binding to the membrane impermeable tracer [^3^H]-NMS, was determined as described above.

### Hypertonic sucrose treatment

HEK-293 cells transfected with hM_1 _receptors were plated onto poly-D-lysine coated 24-well plates on the day after transfection. The cells were incubated for 30 minutes in the presence or absence of hypertonic sucrose (0.4 M) prior to addition of the ligands and an additional incubation for 30 min with the ligands. Cells were then washed with room temperature (RT) PBS, ice cold PBS containing 2 nM [^3^H]-NMS was added and binding carried out for 90 min at 12°C. Cells were removed from the plates, harvested onto 24-well filter plates and washed with cold PBS. Radioactivity was quantitated by scintillation counting using a Top Count scintillation counter.

### Immunofluorescence confocal microscopy

Construction of hM_1 _gene containing the EYMPME (EE) epitope tag (EE-hM_1_) and generation of mouse monoclonal antibody to EE tag were previously described [17,33]. Internalization of this EE-hM_1 _receptor has been well characterized and it has been shown that the N-terminal tag does not interfere with normal trafficking and signaling of the M_1_receptors [17,33].

Transiently-transfected HEK-293 cells expressing EE-hM_1 _receptors were grown overnight on CC2 chamber slides (Nunc Inc., Napperville, IL). Treatment with various concentrations of compounds was carried out at 37°C for 2 hrs. After removing the ligands, cells were fixed for 10 min at RT with 3.7% paraformaldehyde in PBS, and permeabilized in PBS containing 0.25% fish gelatin and 0.04% saponin. Following fixation, cells were labeled with anti-EE monoclonal antibody for 1 h, washed three times with PBS, incubated with Alexa Fluor 488 goat anti-mouse secondary antibody (Invitrogen, Carlsbad, CA) for 30 min in dark, followed by three washes with PBS and one with water. Slides were mounted using Fluoromount G [17,33]. Images were collected on a Delta Vision Optical Sectioning microscope consisting of an Olympus IX-70 microscope (Tokyo, Japan) and a photometrics CH 350 cooled CCD camera. An Olympus oil immersion 60× objective was used to collect the images.

### Receptor selection and amplification (RSAT) assays

Receptor Selection and Amplification (RSAT) functional assays were carried out as described previously [4,5]. Concentration response curves were generated using non-linear regression to fit the data to appropriate logistic equations using GraphPad Prism Software (Graph-Pad Software, Inc., San Diego, CA).

### Phosphatidylinositol (PI) hydrolysis assays

PI hydrolysis assays were performed as described previously with some modifications [4,5]. Exponentially growing CHO cells stably expressing hM_1 _muscarinic receptors (CHO-M_1_) were harvested, plated onto 96-well TC plates in media containing [^3^H]-myo-inositol (2 μCi/ml), and allowed to attach overnight. On the day of the assay, supernatant was removed and the cells washed three times. Fresh serum-free medium containing 10 mM LiCl and varying concentrations of each ligand was added to the cells. After 60 minutes at 37°C, supernatant was removed and 20 mM formic acid was added to the cells. Following 60 minutes at 0°C, a sample of the supernatant was removed and counted in the scintillation counter. Data were analyzed using non-linear regression analysis using GraphPad Prism Software.

### Bioluminescence resonance energy transfer (BRET-2) assay

BRET-2 assays were performed as described [37] with the following modifications. cDNA encoding the human muscarinic M_1 _receptor was cloned into the plasmid pRLuc(h) (Perkin Elmer Life Sciences, Waltham, Massachusetts) to generate a DNA vector for expression of the fusion protein M_1_-Luc (M_1 _carboxyl-terminally tagged with *Renilla luciferase*). Beta-arrestin-1 cDNA was cloned into plasmid DNA pGFP^2 ^(PerkinElmer Life Sciences, Waltham, Massachusetts) to generate a DNA vector for expression of the fusion protein GFP2-β-arrestin-1 (β-arrestin-1 amino-terminally tagged with GFP2). HEK-293T (2 × 10^6^) cells were plated onto 10 cm^2 ^tissue culture plates and cultured for two days. Cells were transiently co-transfected with 1 μg of the bioluminescence donor plasmid encoding M_1_-Luc and 40 μg of the fluorescence acceptor plasmid encoding GFP2-β-arrestin-1 using Polyfect (QIAGEN, Valencia, CA) following manufacturer's instructions. Two days after transfection, cells were harvested and re-suspended at a density of 2 × 10^6 ^- 4 × 10^6 ^cells/ml (depending on transfection efficiency) in PBS pH 7.5, containing glucose and sodium pyruvate. BRET-2 signals were calculated as the ratio between the *Renilla luciferase *emission and the GFP2 emission corrected by the background emissions of non-transfected cells.

### Induction of persistent activation of muscarinic receptors

CHO-M_1 _cells were labeled with [^3^H]-myo-inositol overnight as described above for PI assays. On the day of the assay, the cells were washed three times with PBS and treated for 1 minute with serum free media alone, or medium containing carbachol, AC-260584, xanomeline, AC-42 or NDMC. Basal levels of PI turnover after this 1 minute of treatment with each ligand was measured by washing off the ligand, addition of assay buffer containing 10 mM LiCl_2 _and incubation for an additional 60 minutes at 37°C [38]. Inositol phosphate levels were assessed as described above.

## Competing interests

All authors of this manuscript are past or present employees of ACADIA Pharmaceuticals Inc.

## Authors' contributions

CND contributed to study design and authoring of manuscript, carried out all receptor regulatory, confocal microscopy and PI hydrolysis assays; SRB was responsible for RSAT assays and provided input in study design and revision of the manuscript, HHS performed the BRET-2 assay; MF, KK and B-RT were responsible for synthesis of AC-260584 and its analogues; DWB provided valuable input in the construction and revision of the manuscript; JL conceived of the study, participated in its design and authored the manuscript. All authors have read approved the manuscript for publication.

## Authors' information

CND has published several manuscripts on the signaling of G protein coupled receptors.

JL has over 15 years of experience working with muscarinic receptors, more than 10 years investigating regulatory pathways of various receptors including muscarinic receptors and has published several manuscripts on signaling and regulation of muscarinic receptors.

SRB, JL and DWBspent several years investigating activities of various allosteric and orthosteric agonists of muscarinic receptors.
